# Lack of complex type N-glycans lessens aberrant neuronal properties

**DOI:** 10.1371/journal.pone.0199202

**Published:** 2018-06-14

**Authors:** M. Kristen Hall, Douglas A. Weidner, Austin A. Whitman, Ruth A. Schwalbe

**Affiliations:** 1 Department of Biochemistry and Molecular Biology, Brody School of Medicine, East Carolina University, Greenville, North Carolina, United States of America; 2 Department of Microbiology and Immunology, Brody School of Medicine, East Carolina University, Greenville, North Carolina, United States of America; Universidade de Sao Paulo, BRAZIL

## Abstract

Modifications in surface glycans attached to proteins via *N*-acetylglucosamine-β1-*N*-asparagine linkage have been linked to tumor development and progression. These modifications include complex N-glycans with high levels of branching, fucose and sialic acid residues. Previously, we silenced *Mgat2* in neuroblastoma (NB) cells, which halted the conversion of hybrid type N-glycans to complex type, to generate a novel cell line, NB_1(-*Mgat2*). By comparing the aberrant cell properties of the NB_1(-*Mgat2*) cell line to the parental cell line (NB_1), we investigated the impact of eliminating complex type N-glycans on NB cell behavior. Further, the N-glycosylation pathway in the NB_1(-*Mgat2*) cell line was rescued by transiently transfecting cells with *Mgat2*, thus creating the NB_1(-/**+***Mgat2*) cell line. Changes in the N-glycosylation pathway were verified by enhanced binding of E-PHA and L-PHA to proteins in the rescued cell line relative to those of the NB_1(-*Mgat2*) cell line. Also, western blotting of total membranes from the rescued cell line ectopically expressing a voltage-gated K+ channel (Kv3.1b) revealed that N-glycans of Kv3.1b were processed to complex type. By employment of various cell lines, we demonstrated that reduction of the complex type N-glycans diminished anchorage-independent cell growth, and enhanced cell-cell interactions. Two independent cell invasion assays showed that cell invasiveness was markedly lessened by lowering the levels of complex type N-glycans while cell mobility was only slightly modified. Neurites of NB cells were shortened by the absence of complex type N-glycans. Cell proliferation was reduced in NB cells with lowered levels of complex type N-glycans which resulted from hindered progression through G1+Go phases of the cell cycle. Overall, our results illustrate that reducing the ratio of complex to hybrid types of N-glycans diminishes aberrant NB cell behavior and thereby has a suppressive effect in cell proliferation, and cell dissociation and invasion phases of NB.

## Introduction

Modifications in surface glycans attached to proteins by *N*-acetylglucosamine-β1-*N*-asparagine (GlcNAcβ1-*N*-Asn) linkage is a hallmark of tumor development and progression [1, 2]. All N-glycans have a conserved pentasaccharide, and various branch points of this common core are catalyzed by the *N*-acetylglucosaminyltransferases (GnTs, encoded by *Mgat* genes), resulting in the three major types of *N*-glycans: oligomannose, hybrid, and complex [3]. GnT-I (encoded by *Mgat1*) and GnT-II (encoded by *Mgat2*) are responsible for converting oligomannose type to hybrid type, and hybrid type to complex type, respectively [3]. Further GnT-IV (encoded by *Mgat4*) and GnT-V (encoded by *Mgat5*) increase branching of complex type N-glycans [3]. The highly branched N-glycan structures have been identified in metastatic phenotype, and thus provided the basis for glycan based therapeutics [4]. Initial clinical trials used swainsonine to prevent the conversion of hybrid type N-glycans to complex type [5], and thus reduce the levels of branched N-glycan structure [6–8]. However, trials were halted since the drug was quite toxic [9, 10].

Neuroblastoma (NB), a cancer derived from the sympathetic nervous system, is the most common solid tumor in childhood accounting for about 15% of pediatric cancer related deaths [2]. Although recent advances have improved overall prognosis, long term survival remains poor [2]. Like other types of cancers, altered cell surface glycans have been associated with tumor development and progression, although the repertoire of glycans involved, as well as their roles in facilitating or governing cancer progression is uncertain [1, 2]. It has been shown that higher expression of the *Mgat5* gene (encodes GnT-V) was associated with a more favorable prognosis in neuroblastoma [11]. Another study revealed an incline in several distinct β1,6-branched N-glycans (produced by the action GnT-V) and a decline in some hybrid type N-glycans (produced by decreased activity of GnT-II) in cells derived from high risk neuroblastoma (NLF) relative to cells derived from low risk neuroblastoma (SY5Y) [12]. Other studies have shown that Anaplastic Lymphoma Kinase (ALK), a receptor kinase, was upregulated in advanced/metastatic neuroblastomas [13, 14]. Further, N-glycosylation processing of ALK receptors, and the pro-survival signaling pathway were impaired by treatment of ALK-positive neuroblastoma cells with tunicamycin [15].

Earlier studies from our lab showed that cell behavior was changed by the type of N-glycans at the cell surface in Chinese Hamster Ovary (CHO) cells [16–18]. Further, we engineered and characterized a NB cell line with *Mgat2* silenced, which resulted in a cell line that is unable to convert hybrid to complex types of N-glycans [19]. Herein, studies in the parental and N-glycosylation mutant NB cell lines [19], as well as the rescued N-glycosylation mutant NB cell line, were conducted to elucidate whether a lowered ratio of complex to hybrid types of N-glycans could diminish or promote aberrant tumor cell properties in NB. Results of this innovative study support that a lowered ratio of complex to hybrid types of N-glycans in NB cells suppresses cell proliferation, and cell dissociation and invasion phases in neuroblastoma.

## Materials and methods

### Cell lines, cell culture and cell transfection

Rat B35 neuroblastoma (NB) cells were purchased from American Type Culture Collection (Manassas, VA, USA) and used to generate the NB_1 and NB_1(-*Mgat2*) cell lines, and also, various stable NB cell lines expressing wild type (WT) or N220/9Q (DM) Kv3.1b proteins, as we previously described [19]. NB cell lines were cultured and maintained in DMEM containing 10% FBS, 50 U/mL penicillin and 50 μg/mL streptomycin at 37° in a 5% CO_2_ atmosphere. NB_1(-*Mgat2*) cell line was rescued by transient transfection with pCMVSport6 recombinant vector coding the mouse Mgat2 cDNA (Thermofisher Scientific, MA, USA) using the Lipofectamine^®^ 2000 (Thermofisher Scientific, MA, USA) protocol [18].

### Western and lectin blots

Total cell membrane and whole cell lysate samples for western and lectin blotting, respectively, were electrophoresed using 10% SDS gels for 1.7 h at 20 mA [16, 18]. In brief, separated proteins were blotted to PVDF membranes (Millipore, MA, USA) for 2.5 h at 250 mAmps. Blotted membranes were incubated and developed. Unglycosylated and glycosylated Kv3.1b proteins were detected using the mouse anti-Kv3.1b antibody (Neuromab, CA, USA). Biotin-conjugated *Phaseolus vulgaris* Erythoagglutinin (E-PHA) or *Phaseolus vulgaris* Leucoagglutinin (L-PHA) (Vector Laboratories, CA, USA) was employed to probe membranes containing separated glycosylated proteins. Images were acquired using Kodak gel logic 100 imaging system.

### Anchorage-independent growth

The ability of cells to grow as anchorage-independent colonies was assayed via the soft agar assay [20]. Low melting temperature agarose (1%) in DMEM supplemented with 10% FBS was aliquoted into a 6 well plate and allowed to solidify for 30 minutes at room temperature to form the base layer. Equal parts of cell suspension mixed with 1% low melting noble agar was added to the top of the solidified base layer (~6,000 c/well). The cells were cultured for 13 days. Images were acquired using a 4X objective on an Olympus IX73 microscope. ImageJ software was utilized to measure area of the cell colonies and number of cell colonies.

### Dissociation assays

Cells were seeded on 35 mm CellBind culture dishes (Corning, NY, USA) and allowed to grow to confluence for 2 days [21]. In short, cells were rinsed twice with media and re-suspended in serum free media. Cells were detached by one complete rotation with a cell scraper. Detached cells were dissociated by pipetting ten times with a 1 mL pipet tip. Images (25–30 fields/dish) were acquired on an Olympus IX 71 microscope using a 10X objective. Area of cell aggregates (≥ 10 cells/aggregate) were measured using Image J software.

### Cell invasion assay

Cell invasion was assayed using the BD Falcon matrigel invasion chambers (BD Biosciences, CA, USA). The assay was performed according to manufacturer’s instructions. In brief, DMEM was added to the transwell inserts in 24 well plates for 2 hours at 37° to rehydrate the matrigel. Media was removed and 2.5 X 10^4^ cells in 500 μl of serum free DMEM were seeded in each transwell insert. Quadruplicate samples were used for each of the three experiments. The lower chamber of the plate was filled with 500 μl of NIH-3T3 conditioned media. After 24 hour incubation at 37°, the cells remaining on the interior of the transwell insert were gently removed, while the invasive cells on the bottom surface of the insert were fixed with 100% methanol and stained with 1% Toluidine blue. The membranes were removed from the insert and cells from five fields per membrane were counted using a Nikon TMS microscope. Images were acquired using an Olympus IX73. The number of invasive cells was determined for each cell line and then normalized to the NB_1 cell line.

### Wound healing assays

Cell migration experiments were conducted as previously described [22]. Cells were seeded and allowed to grow to confluence, at which time the media was removed and wounds were made in the cell monolayer using a beveled 200 μl pipet tip. Cells were rinsed twice with media to remove floating cells and images were obtained at 0 and 19 h on an Olympus IX 71 microscope using a 4X objective. The average wound closure (AU) was determined by taking the difference in wound closure between the initial width and final width of the wound.

### 3D spheroid assay

The cancer cell spheroid assay was similar to that as illustrated [23]. Spheroids were formed by seeding cells (5 X 10^5^ c/mL) in 30 μl drops onto the lid of a 100mm culture dish and allowed to incubate for 96 h at 37° in the hanging drop. Drops were collected and placed in a microcentrifuge tube and the spheroids were allowed to settle for 10 minutes. Spheroids were aspirated from the media pool and combined with 100 μl of Matrigel (Fisher Scientific, GA, USA). Using pre-chilled pipet tips, 40 μl of spheroid/Matrigel mixture was pipetted into the center of the wells of a 24-well plate (4 wells per cell line). Plate was placed at 37° for 30 minutes to allow polymerization of culture. Subsequently, 1 mL of media was added to culture, and cultures were incubated for 24 hrs. Images were acquired using a 10X objective on an Olympus IX73 microscope. The sphere area and invasive area were measured using ImageJ software. Invasion was calculated by dividing invasive area by sphere area.

### Cell growth assay

Cells were seeded in triplicate in 12 well culture dishes (Corning, NY, USA) at 1 X 10^4^ cells per well and allowed to incubate for 48h, 72h, and 96h. Cells were trypsinized and counted by hemacytometer using a Motic AE31 light microscope (Ted Pella, Inc., CA, USA). Experiment was conducted four times and the mean of triplicates per experiment was utilized to calculate fold increase per day.

### Bromodeoxyuridine cell proliferation assay

The proliferation of cells was determined by the 5-bromo-29 deoxyuridine (BrdU) proliferation assay (Millipore, MA, USA) following the manufacturer’s protocol. In brief, 2 X 10^4^ cells were seeded in 100ul in a 96 well plate and incubated for 2 hours at 37°. BrdU was added and allowed to incorporate with the cells for 24 hours. Cells were then fixed for 30 minutes at room temperature. Next, addition of anti-BrdU monoclonal antibody for 1 hour was followed by secondary goat-anti mouse IgG peroxidase conjugate for 30 minutes at room temperature. Cells were then incubated with peroxidase substrate for 30 minutes at room temperature and quantified by measuring the absorbance at 450 nm using a Multiskan FC plate reader (Fisher Scientific, GA, USA).

### Cell cycle assay

Cell cultures were synchronized by plating cells onto 60mm culture dishes in DMEM with 10% FBS (MediaTech, VA, USA) and allowed to grow to similar confluence. Media was then removed and replaced with serum free media for 24 hours, at which point some of the plates were harvested for the 0 hour time point and the remaining plates were refed serum and incubated an additional 21hrs before harvesting. In both cases, cells were harvested by the addition 0.05% Trypsin-EDTA (Life Technologies, CA, USA), followed by collection of the cell suspension and centrifugation at 100 x g for 5 minutes at 4°C in an Eppendorf 5417R centrifuge. Following a wash with PBS, the cells were fixed with 70% Ethanol and placed at -20°C for at least 20 hours. Cells were pelleted, ethanol was removed and the cells were washed once with PBS. Propidium Iodide staining solution (50 μg/ml PI, 20 μg/ml RNase A) was added and allowed to incubate for 30 minutes at 37°C. Subsequently, cells were analyzed using a FACS Vantage flow cytometer (Becton Dickinson Biosciences, CA, USA) using 488 nm laser excitation and emission centered at 600 nm. Cell Quest software was utilized to measure cell cycle distribution in the G1, G0+G1, S, and G2+M phases.

### LDH cytotoxicity assay

Cell cytotoxicity was assayed using the Pierce LDH Cytotoxicity Assay Kit (Pierce Biotechnology, IL, USA). Cells of similar confluency were seeded in DMEM onto 96 well culture dishes and allowed to incubate overnight at 37°C. Next, lysis buffer or sterile water (control) was added to duplicate wells and incubated for 45 min at 37°C. Subsequently, sample media (50μl) was transferred to a 96 well dish and incubated with supplied substrate. After 30 min incubation, stop reagent was added to complete the reactions and absorbance was read at 450 nm. The average OD at 450 nm was calculated from duplicate wells from two separate experiments.

### MTT assay

Chemotherapeutic drug cytotoxicity was assayed via the MTT assay. Cells were seeded at 2.5 X10^5^ c/mL in DMEM in a 96 well plate and allowed to incubate 2 hrs for cells to attach. Chemotherapeutic drugs, Cisplatin (25μg/mL) and doxorubicin (25μg/mL), were added in triplicate and allowed to incubate for 72hrs at 37°C 5% CO_2_. Thereafter, (5mg/mL) of 3-[4,5-dimethylthiazol-2-yl]-2,5-diphenyltratrazolium bromide (MTT) (Calbiochem, Burlington, MA) was added to all wells and allowed to incubate for 4 hrs, followed by the addition of solubilization solution to dissolve the formazan crystals. Absorbance was measured at 560nm using a Multiskan FC plate reader (Fisher Scientific, Atlanta, GA). The % cell viability was calculated as follows:

$$\%\;viability=\frac{absorbance\;of\;experimental\;well}{absorbance\;of\;control\;well}X\;100.$$

### Data analysis

Image J software was used to measure the areas of cell clusters, colonies, spheroids, and spheroid invasion. Adobe photoshop was used for construction of Figs of lectin and western blots, and also, wound size measurements. Origin 7.5 was used for graphics and statistics. Data is presented as the mean ± S.E. where *n* denotes the number of observations. The unpaired student’s t-test was utilized when comparing two populations, and one-way ANOVA with Bonferroni adjustments when more than two populations were tested.

## Results

### GnT-II restored the N-glycosylation pathway in the engineered glycosylation mutant cell line

**Fig 1:**
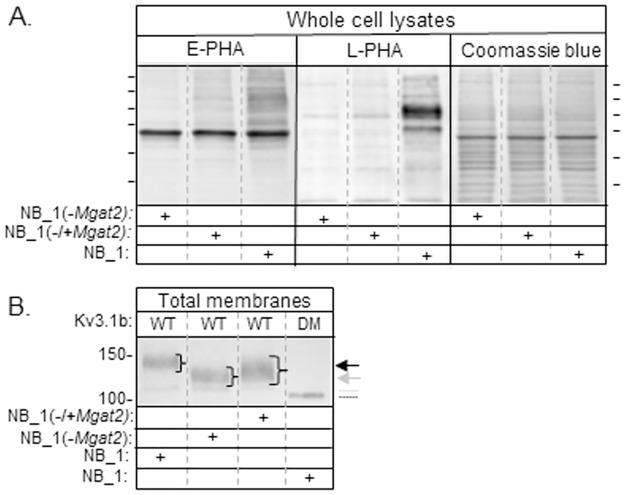
Changes in the N-glycosylation pathway due to expression of *Mgat2* in NB cells. A lectin blot of whole cell lysates from NB cells with (NB_1), and without *Mgat2* (NB_1(-*Mgat2*)), and also, the NB_1(-*Mgat2)* cell line transiently transfected with *Mgat2* to create the rescued cell line, called NB_1(-/+*Mgat2*) *(A)*. Proteins separated on membranes were probed with *Phaseolus vulgaris* Erthroagglutinin (E-PHA) or *Phaseolus vulgaris* Leucoagglutinin (L-PHA). Coomassie blue stained SDS gels reflect similar amounts of protein per lane. Lines adjacent to blot and gel indicate molecular weight standards in kDa: 250, 150, 100, 75, 50, and 37 from top to bottom. Western blot of total membranes from NB_1, NB_1(-*Mgat2*), and NB_1(-/+*Mgat2*) cell lines transfected with glycosylated (WT) and unglycosylated (DM) Kv3.1b *(B)*. (+) indicates the cell line examined. Black and gray arrows represent complex and hybrid types of N-glycans, respectively, attached to Kv3.1b. The gray dotted line indicates oligomannose type glycans attached to Kv3.1b, and the black line denotes unglycosylated Kv3.1b. Molecular weight markers adjacent to the membranes are in kDa.

Previously, we engineered a neuroblastoma (NB_1) cell line with *Mgat2* silenced, called the NB_1(-*Mgat2*) cell line[19]. Lectin binding studies revealed predominant expression of hybrid type N-glycans, and the absence of complex N-glycans, including bisecting complex in the NB_1(-*Mgat2*) cell line [19]. The NB_1 cell line is a clonal cell line created from the rat B35 neuroblastoma (NB) cell line [19]. Differences between the N-glycans for NB and NB_1 were undetected [19]. To further confirm that the type of N-glycans on the cell surface of the N-glycosylation mutant cell line was due to the absence of GnT-II activity, lectin blotting was conducted with whole cell lysates from NB_1 and NB_1(-*Mgat2*) cell lines, and also, the NB_1(-*Mgat2*) cell line transiently transfected with *Mgat2*, referred to as NB_1(-/+*Mgat2*) (Fig 1A). E-PHA was used since it has a much higher affinity for complex type N-glycans with bisecting N-acetylglucosamine than hybrid type N-glycans with bisecting N-acetylglucosamine [24]. L-PHA was employed to detect complex type N-glycans [24]. In both cases, numerous bands of high intensity were observed in the lane containing the NB_1 sample while the intensity of the bands was greatly reduced in the lane with NB_1(-*Mgat2*). When the NB_1(-*Mgat2*) cell line was transiently transfected with *Mgat2*, the band intensity markedly increased relative to those of the nontransfected NB_1(-*Mgat2*) cell line. These results indicate that complex type N-glycans, including complex type N-glycans with bisecting N-acetylglucosamine, were expressed by transfected cells. The Coomassie blue stained gel showed that the levels of protein loaded per well for the lectin blot were quite similar.

To date, we showed that the N-glycans attached to wild type (WT) Kv3.1b heterologously expressed in NB_1 cells, as well as NB cells, were of complex type while those of WT Kv3.1b in NB_1(-*Mgat2*) cells were of hybrid type [19]. The electrophoretic mobility of WT Kv3.1b protein in the NB_1(-*Mgat2*) cell line transiently transfected with *Mgat2* was detected by Western blotting, and compared to those of WT Kv3.1b in NB_1 and NB_1(-*Mgat2*) cell lines, as well as unglycosylated (DM) Kv3.1b in the NB_1 cell line (Fig 1B). The unglycosylated Kv3.1b protein has both N-glycosylation sites removed by mutating Asn220 and Asn229 to Gln residues [25]. As expected, the Kv3.1b protein with complex type N-glycans migrated significantly slower than that with hybrid type N-glycans, and much slower than its unglycosylated counterpart [19]. The faint immunoband in samples expressing WT Kv3.1b represents the Kv3.1b with oligomannose type N-glycans [22]. In all cases, the Kv3.1b immunoband observed from NB_1(-/+*Mgat2*) cells was much more diffused and overlapped with the electrophoretic positions of Kv3.1b with complex N-glycans, and that of hybrid N-glycans. Taken together, our results indicate that the N-glycans in NB_1(-*Mgat2*) cells could be converted to complex type by ectopic expression of GnT-II, and thus alterations in the N-glycosylation pathway of the NB_1(-*Mgat2*) cell line could be restored.

### Lack of complex type N-glycans reduce the ability of neuroblastoma cells to form tumors

**Fig 2:**
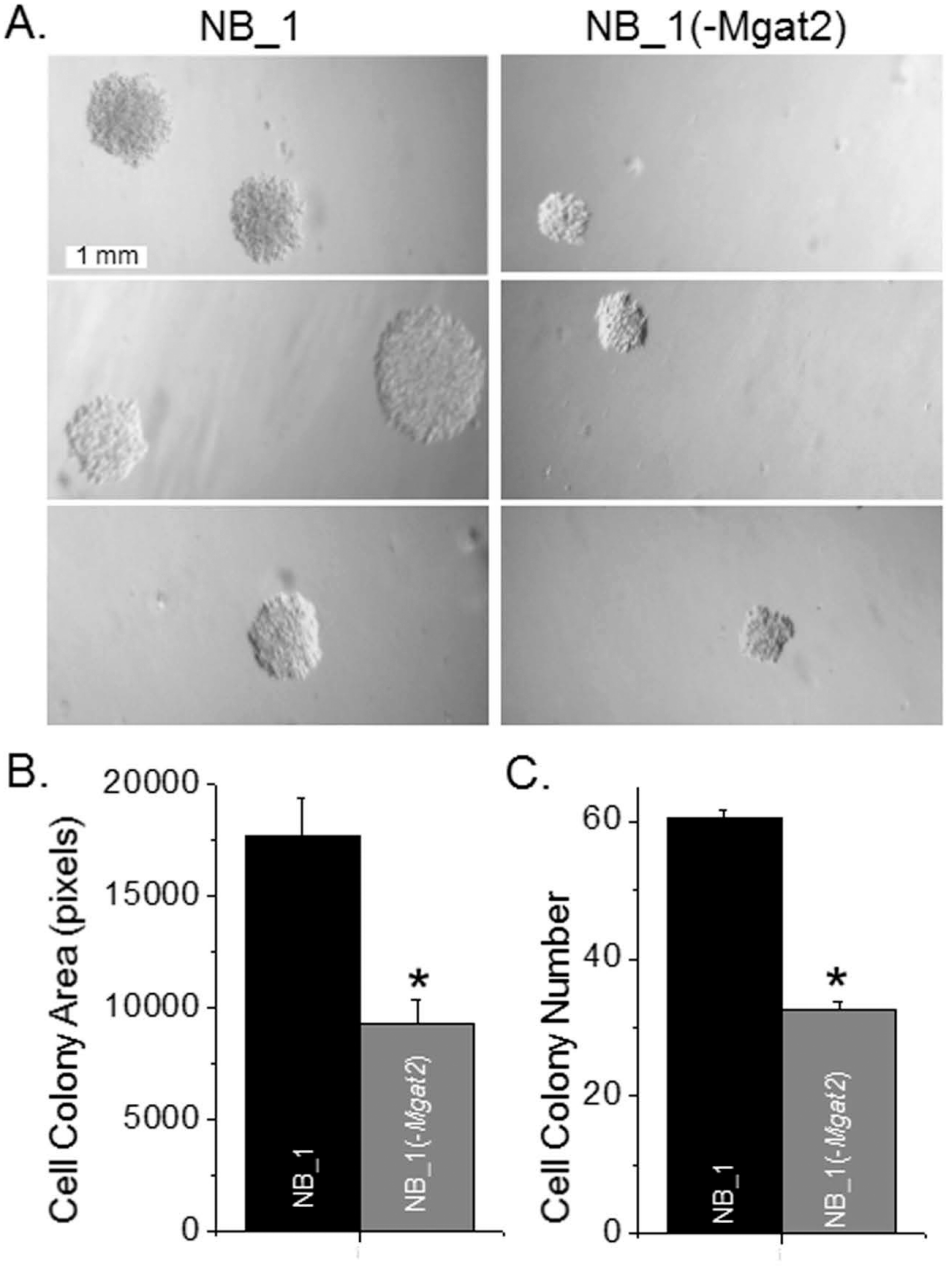
Lack of *Mgat2* expression in NB cells reduces tumorigenic potential. Representative DIC images of cell colonies from NB_1 (left panels) and NB_1(-*Mgat2*) (right panels) cell lines obtained from the anchorage independent cell growth assay conducted on 3 separate occasions *(A)*. Mean values of cell colony area *(B)* from at least 98 colonies, and cell colony number *(C)* per experiment. *P<0.01.

To determine the influence of complex type N-glycans on the ability of the NB cells to proliferate without attachment to or spreading onto a substratum, we conducted an anchorage-independent cell growth assay. After 13 days of cell growth, micrographs were obtained of cell colonies from 3 wells for each of the 3 experiments. Representative micrographs of the size and number of cell colonies are shown (Fig 2A). NB cells expressing complex type N-glycans (NB_1) formed significantly larger cell colonies (Fig 2B), and a greater number of cell colonies (Fig 2C) than NB cells lacking complex type N-glycans (NB_1(-*Mgat2*)), indicating that complex type N-glycans contribute to tumorgenicity of NB cells.

### Reduced levels of complex type N-glycans enhance cell-cell adhesion

**Fig 3:**
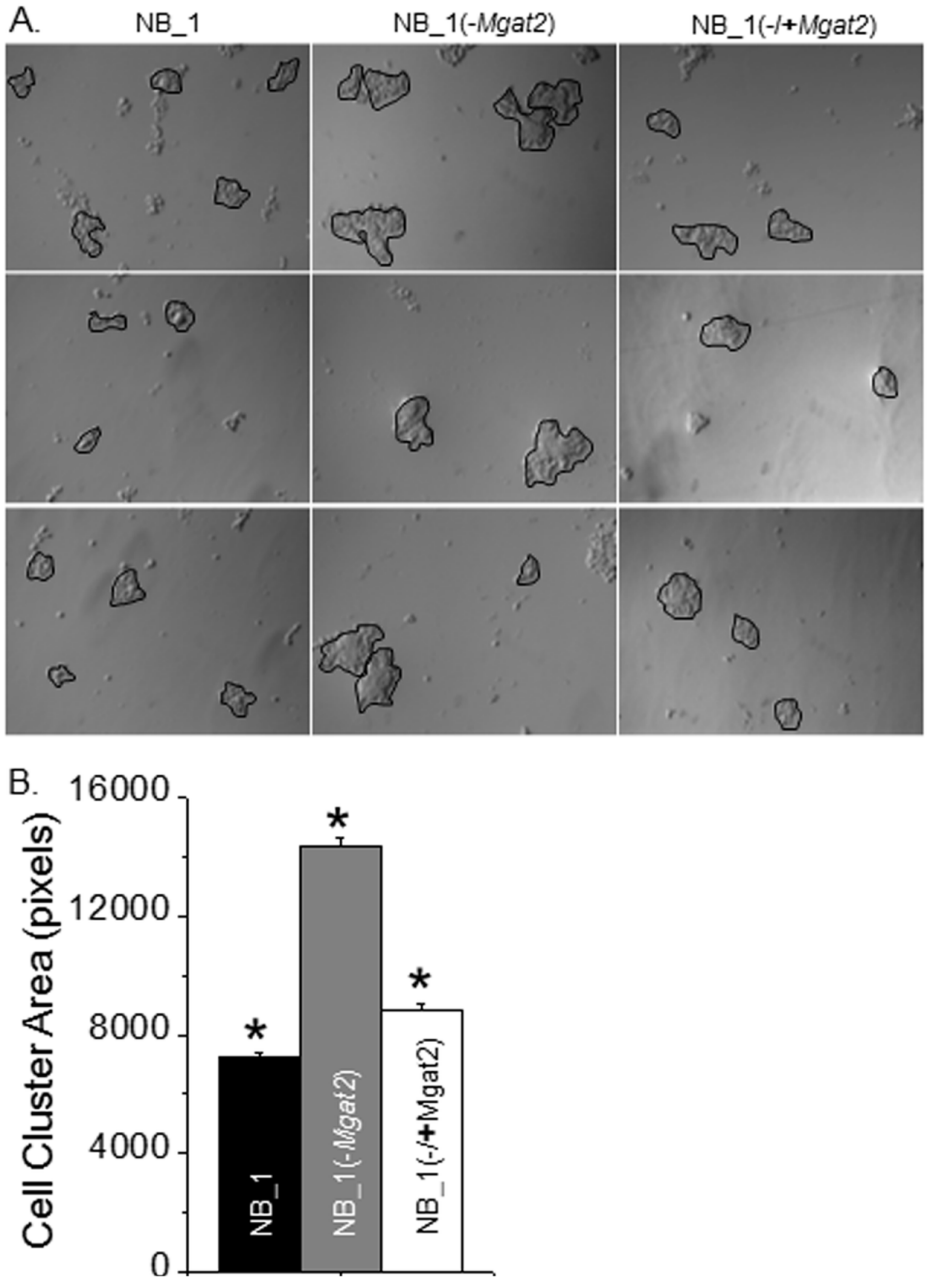
Reduced *Mgat2* expression in NB cells enhances cell-cell adhesion. Representative DIC images acquired from 3 cell dissociation experiments of NB_1 (left panels), NB_1(-*Mgat2*) (middle panels), and NB_1(-/+*Mgat2*) (right panels) cell lines *(A)*. Cell clusters of more than 10 cells, as encircled in black, were examined. The bar graph represents the mean area of cell clusters for each cell line *(B)*. *P<0.01.

To investigate whether the lessened tumorigenicity correlated with strengthened cell-cell interactions, the cell dissociation assay was employed. Cells were cultured until fully confluent and then cells were detached. Subsequently, the detached monolayer fragments were dissociated by pipetting up and down 10 times. Representative images of the monolayer fragments show that the size of the cell clusters from the NB_1(-*Mgat2*) cell line were larger than those from the NB_1 cell line (Fig 3A), as well as the NB cell line (not shown). Further, when cells lacking *Mgat2* expression were transiently transfected with *Mgat2* (NB_1(-/**+***Mgat2*), the area of the cell clusters were lessened. Quantitative analysis of the clusters from numerous fields of three independent experiments demonstrated that the cell cluster area was increased by about 2-fold when Gn-TII activity was absent (Fig 3B). Subsequently, when GnT-II was reintroduced into NB_1(-*Mgat2*) cells, the mean cell cluster area markedly decreased, and it was only 1.2-fold greater than that of the NB_1 cell line. The clonal NB_1 cell line was not significantly different from the parental NB cell line (not shown). These results revealed that the absence of complex type N-glycans in the NB_1(-*Mgat2*) cell line strengthened the cell-cell interaction, and furthermore that this strengthened interaction could be reduced by reintroduction of GnT-II.

### Cell invasion was decreased by the absence of complex type N-glycans

**Fig 4:**
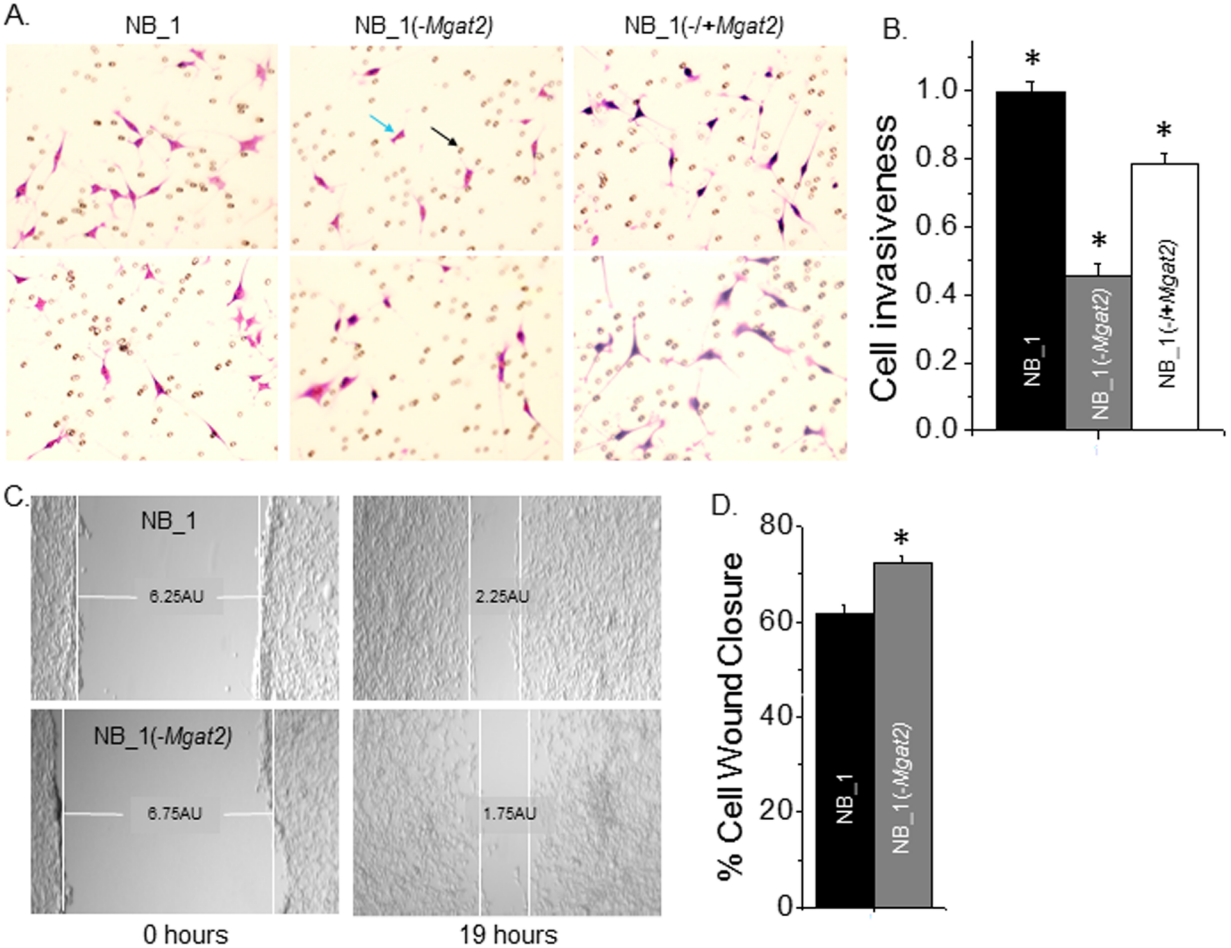
Cell invasiveness is decreased by lowered expression of *Mgat2*. Selected micrographs acquired from matrigel invasion assay of NB_1 (left panels), NB_1(-Mgat2) (middle panels), and NB_1(-/+Mgat2) (right panels) (*A*). Light blue and black arrows denote an invasive cell and a pore in the membranes, respectively. The bar graph represents the number of invasive cells per well from the indicated cell lines normalized to that of the NB_1 cell line (*B*). Cell invasiveness was determined from three experiments performed in quadruplicate for each cell line. *P<0.01. Images of wound healing assay were taken for the NB_1 (upper panel) and NB_1(-Mgat2) (lower panel) at 0 and 19 hr (*C*). White lines at the edge of the monolayer, along with the horizontal line connecting these two vertical lines, are used to show the width of the cell wound. AU represents the arbitrary unit. The bar graph indicates the percent of wound closure in 19 hours from at least 38 wounds obtained on 6 occasions for the respective cell lines *(D)*. *P<0.06. Cell mobility of NB was not significantly different to NB_1.

To determine the effect of complex type N-glycans on cell invasiveness, NB cells were seeded in a chamber insert of the transwell plate, and a chemoattractant was added to the chamber for 24 h under cell culturing conditions. The chemoattractant was medium isolated from NIH3T3 cell cultures. Representative images showed that the NB_1 cell line had higher amounts of cells that migrated through the Matrigel^®^ towards the chemoattractant than the NB_1(-*Mgat2*) cell line (Fig 4A). Further, reintroduction of *Mgat2* expression in the latter cell line encouraged more cells (NB_1(-/**+***Mgat2*)) to migrate through the Matrigel^®^. The number of invasive cells per well were determined in four wells from three separate experiments, showing that NB_1 cells were more invasive than NB_1(-/**+***Mgat2*) cells, and much more invasive than NB_1(-*Mgat2*) cells (Fig 4B). Further, the clonal NB_1 cell line was not significantly different from the parental NB cell line (not shown).

Cell wound healing assays were used to evaluate cell migratory rates of NB cells with different types of N-glycans. Scratches were introduced into monolayers of NB_1, and NB_1(-*Mgat2*) cell lines and images of cell wounds were acquired at 0 h and 19 h under cell culturing conditions (Fig 4C). These images represent one scratch obtained for each cell line from five independent experiments. The percent of cell wound closure was slightly faster for NB_1(-*Mgat2*) cells compared to NB_1 cells, as well as NB cells, indicating that the cell migratory rates were only slightly increased by elimination of complex type N-glycans (Fig 4D). Although the cell migratory rate for NB_1(-*Mgat2*) cells was slightly enhanced, cell invasiveness was markedly diminished by lowered levels of complex type N-glycans.

**Fig 5:**
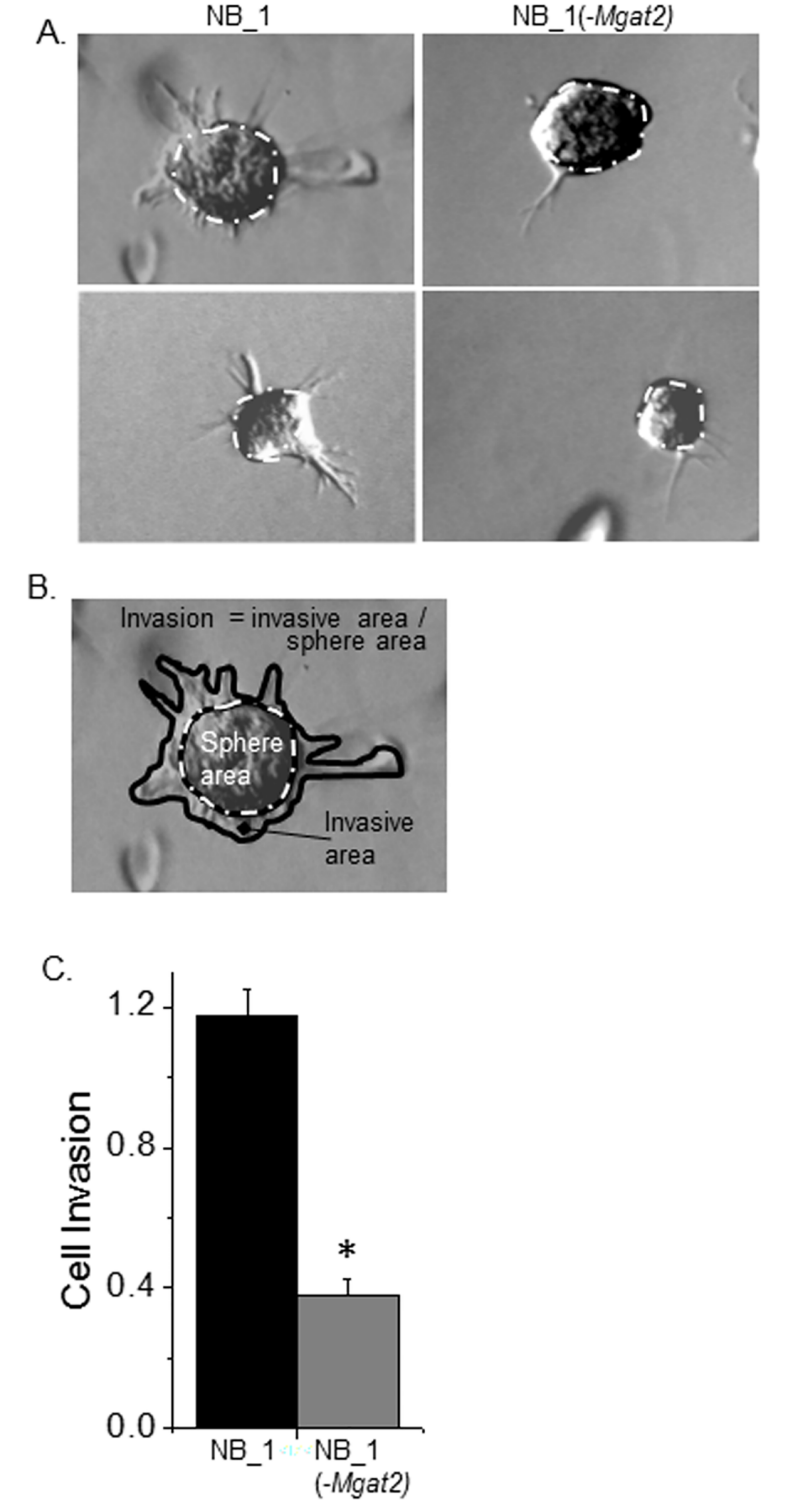
Silencing of *Mgat2* minimizes cell invasiveness of 3D spheroids derived from NB cells. Selected micrographs of spheroids after 13 days of invading into matrix from NB_1 and NB_1(-*Mgat2*) (*A*). Cell invasion was calculated by dividing invasive area by the sphere area (*B*). The invasive area is between the inner and outer black traces. The white dotted line overlaid on the inner black line encircles the sphere area. The bar graph represents the cell invasion of at least 130 spheroids from 3 experiments conducted with the NB cells (*C*). *P<0.00001.

To further support that the absence of complex type N-glycans made NB cells less invasive, the three-dimensional tumor spheroid invasion assay was employed. Spheroids formed by the hanging drop method were cultured in dishes containing Matrigel^®^. After 24 hours of growth, micrographs showed that the invasive area of spheroids for NB_1 cells was larger than that of NB_1(-*Mgat2*) cells (Fig 5A). Cell invasion was determined by measuring sphere and invasive areas, and subsequently dividing the invasive area by sphere area (Fig 5B). The data is summarized from 3 experiments of at least 130 spheroids for each cell line showing that cell invasion for the NB_1 cell line was at least 3-fold greater than NB_1(-*Mgat2*) cell line (Fig 5C). Taken together, these results strongly support that the absence of complex type N-glycans make NB cells less invasive, and furthermore cell invasiveness could be intensified by reintroduction of complex N-glycans.

### Prevention of complex type N-glycans alters cell morphology

**Fig 6:**
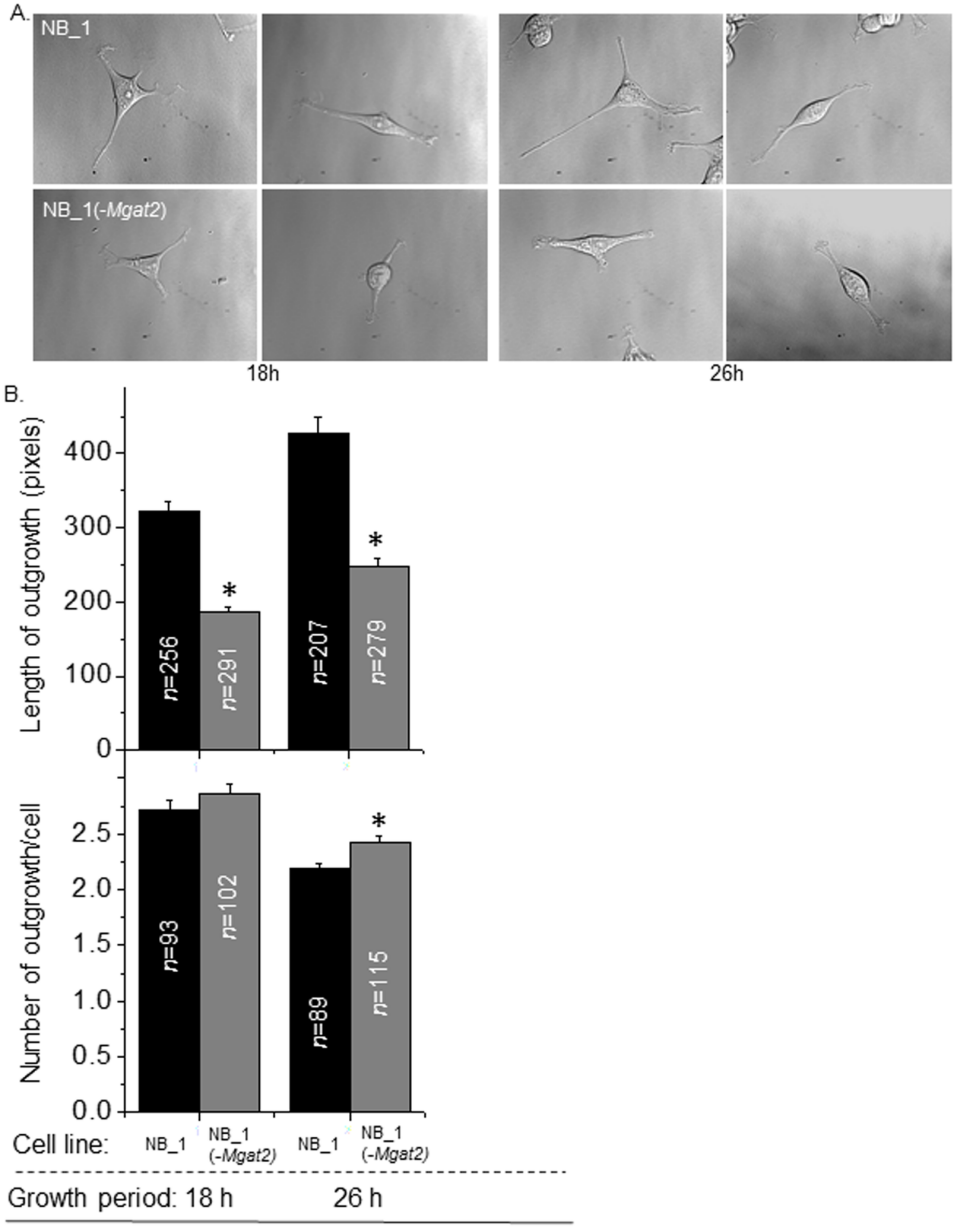
Cell morphology of NB cells are altered by silencing *Mgat2*. Adhered cell growth of the NB_1 and NB_1(-*Mgat2*) cell lines were acquired at different time periods, 18 and 26 h, as shown in the representative DIC images (*A*). Bar graphs show the length of outgrowths (upper panel), and number of outgrowths per cell (lower panel) at both time points (*B*). The experiment was conducted 3 times and at least 207 outgrowths and 93 cells were analyzed. *P<0.01.

To determine if the absence of complex type N-glycans modifies cell morphology, differential interference contrast (DIC) images of NB_1 and NB_1(-*Mgat2*) cells were acquired at different time periods (18 and 26 h) (Fig 6A). The images acquired from three independent experiments suggested that the morphology of the NB_1 cell line was more neuronal-like since the outgrowths (neurites) appeared longer than those of the NB_1(-*Mgat2*) cell line. This difference was proven by measuring the length of outgrowths after cells were cultured for 18h and 26 h (Fig 6B, top panel). As expected, the outgrowths lengthened with longer periods of growth. However, the length of the outgrowths for NB_1(-*Mgat2*) continued to lag relative to those of NB_1. In fact, the outgrowths of NB_1(-*Mgat2*) were 58% of NB_1 at both adhered cell growth periods. The number of outgrowths reduced with adhered growth time, and only some cells had more outgrowths for NB_1(-*Mgat2*) than NB_1 at the later time point (Fig 6B, bottom panel). In all cases, the parental NB cell line was much like the clonal NB_1 cell line (not shown). Thus, the cell morphology of the NB_1(-*Mgat2*) cell line was markedly different from the NB and NB_1 cell lines, indicating that the processing of N-glycans to complex type enhanced the lengthening of neurites.

### Lack of complex N-glycans pslows cell growth and cell cycle progression

**Fig 7:**
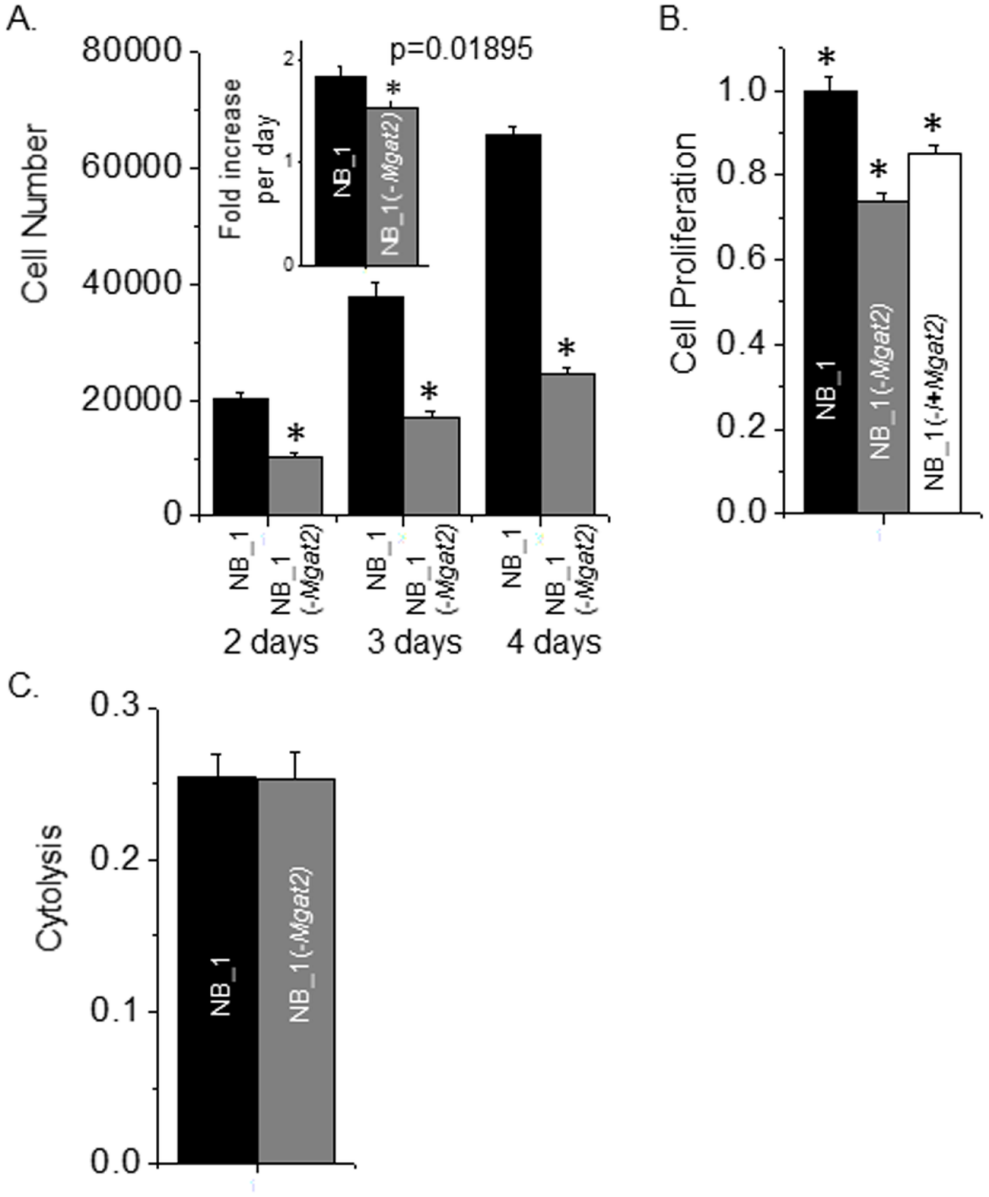
Cell growth and proliferation of NB cells were slowed by lowering the expression of *Mgat2*. The bar graph represents the number of cells for NB_1 and NB_1(-*Mgat2*) at 2, 3, and 4 days of growth from 4 experiments performed in triplicate (*A*). The inset, shows fold increase per day. *P<0.02. The cell proliferation assay were conducted 3 times in triplicate for the various NB cell lines (*B*). *P<0.01. Cytolysis was measured using the LDH assay for NB_1 and NB_1(-*Mgat2*) cells (*C*). No significant difference was observed at *P*<0.05.

Cell growth was followed over a course of four days by plating cells at 15–20% cell confluency. The low density was used to reduce the influence of cell-cell contact on growth rates (Fig 7A). The number of cells at two, three, and four days were considerably lower for NB_1(-*Mgat2*) than NB_1. Although similar numbers of cells were seeded at 0 h, a longer lag time in growth was observed for NB_1(-*Mgat2*) cells relative to NB_1 cells, as indicated at two days of growth time. After this recovery period of adherent growth, NB_1(-*Mgat2*) cells continued to display a slower growth rate than NB_1 cells (Fig 7A, inset). To correlate the changes in cell growth with cell proliferation, incorporation of BrdU into the DNA during its replication process was determined in cells on plates at about 75% confluency. DNA replication occurred at a reduced rate in NB_1(-*Mgat2*) compared to NB_1 (Fig 7B). Further when the glycosylation mutant cell line was transiently transfected with Mgat2 cDNA the replication of genomic DNA was enhanced. The rates of cytolysis for NB_1 and NB_1(-*Mgat2*) were similar (Fig 7C), suggesting that the decrease in cell growth was not due to cell death. Cell growth and proliferation of parental NB cell line was not significantly different from the clonal NB_1 cell line (not shown). Thus, these results showed that lowered levels of complex type N-glycans in NB cells lessened their ability to adapt to adhered growth, and also, it markedly reduced their ability to grow and proliferate.

**Fig 8:**
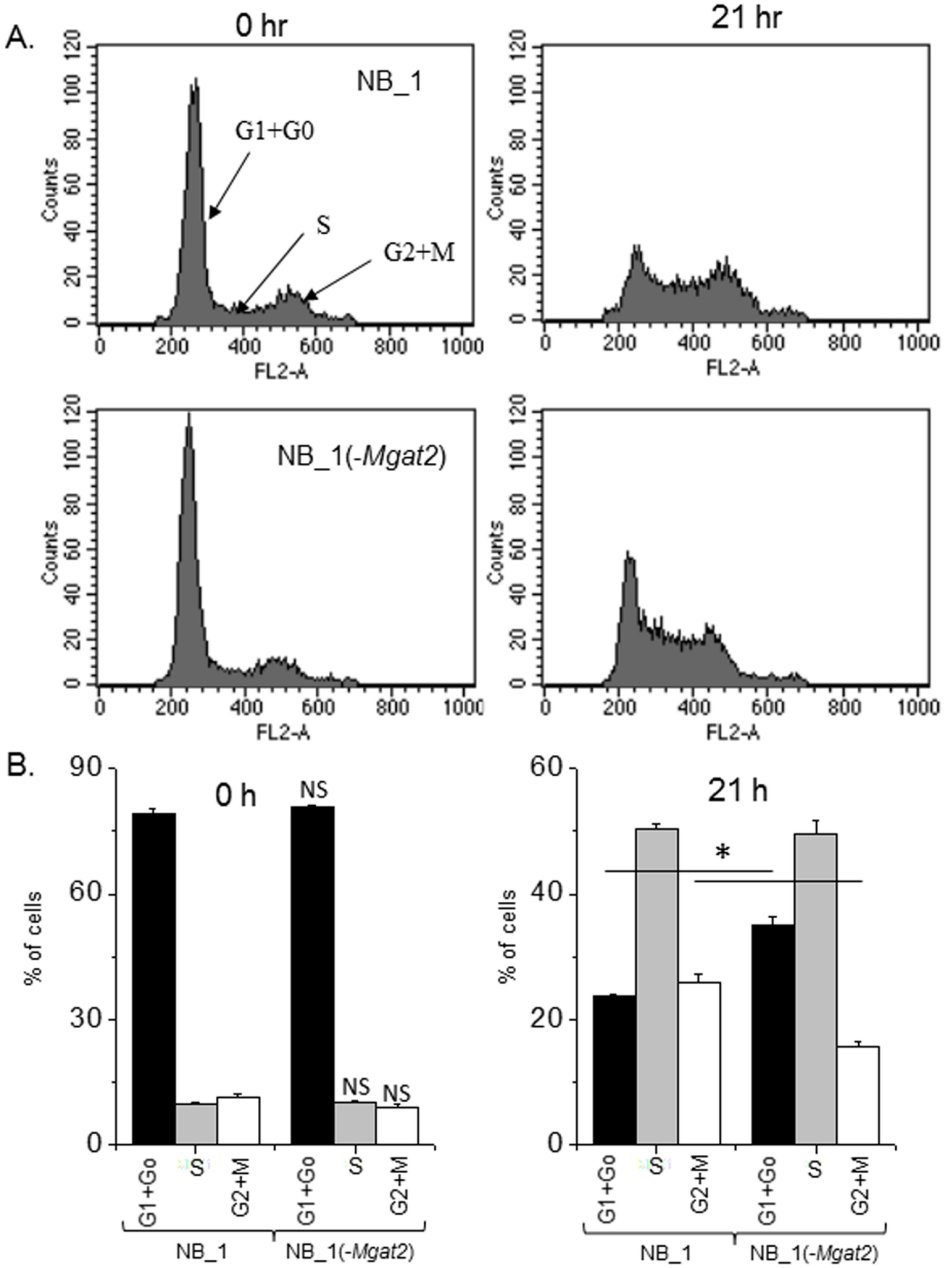
Population of NB cells in the various phases of cell cycle are altered by eliminating *Mgat2* expression. Representative flow cytometry plots of NB_1 and NB_1(-*Mgat2*) cells after serum starvation, 0 hr, followed by addition of serum for 21 hr (*A*). Bar graphs reveal the percent of cells in a given phase of the cell cycle, G1+G0, S, G2+M, at each time point, 0 and 21 hr (*B*). *P<0.05.

To determine if the slowed growth rate of NB_1(-*Mgat2*) cells relative to NB_1 cells correlated with changes in cell cycle progression, we performed flow cytometry analysis of these cell lines at various time points following synchronization of their growth. After incubation of cells in serum free media for 24 h, synchronized cells were grown in serum containing media for 0 and 21 h, and then propidium iodide stained cells were analyzed by flow cytometry. Representative tracings showed similar distributions of cells in the various phases of the cell cycle for NB_1 and NB_1(-*Mgat2*) at 0 h while the distribution was quite different at the 21 h (Fig 8A). Further analysis revealed a significant increase and decrease in the level of NB_1(-*Mgat2*) cells in the G1+Go phase and G2+M phase, respectively (Fig 8B). Taken together, the results indicate that lack of GnT-II expression reduced the growth rate of NB cells, and furthermore the reduction in cell proliferation of NB_1(-*Mgat2*) relative to NB_1 was due to hindering the progression of NB_1(-*Mgat2*) cells through the G1+Go phase of the cell cycle.

**Fig 9:**
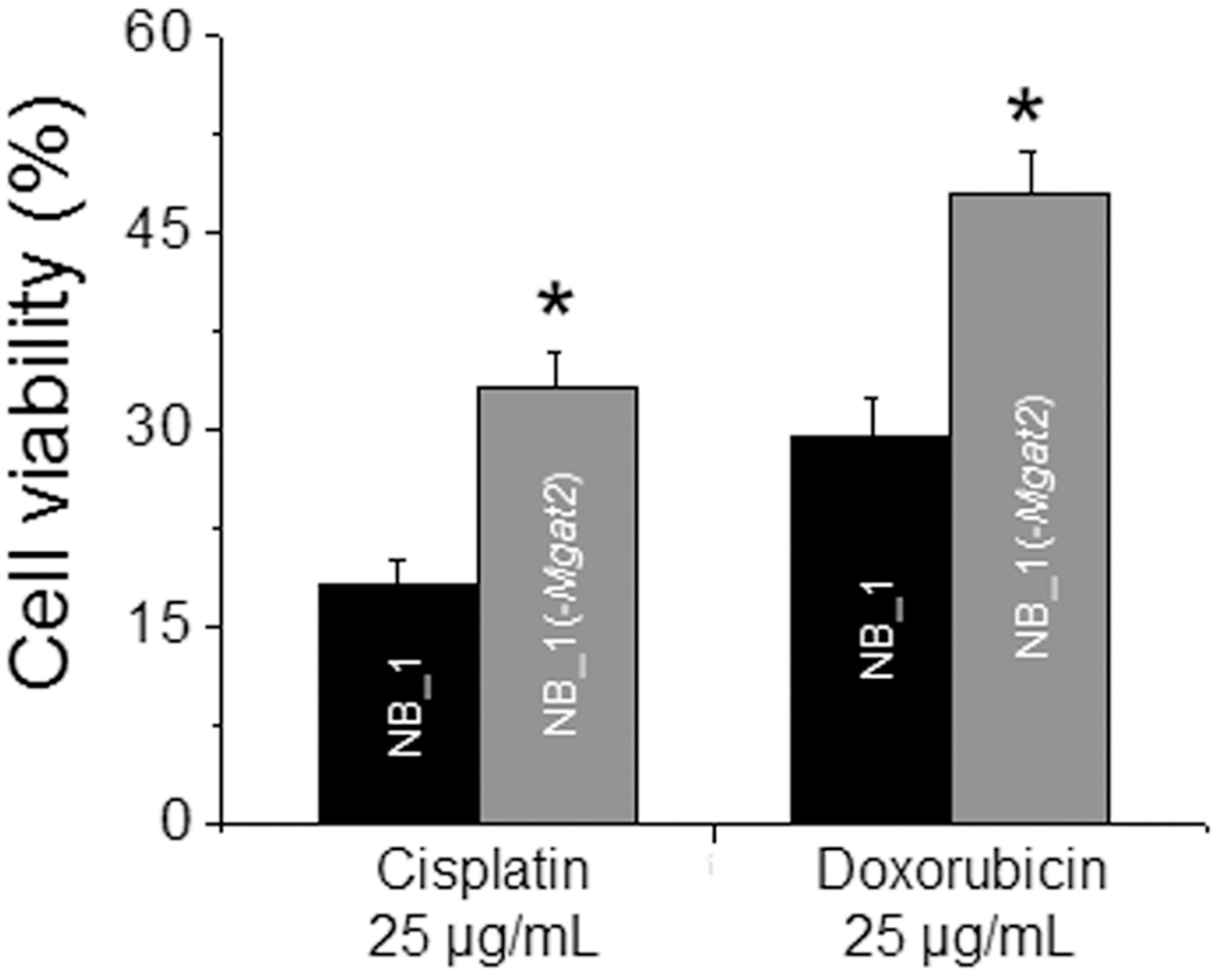
NB cells with *Mgat2* silenced are less sensitive to chemotherapy drugs. The percent of viable cells after treatment of cells with cisplatin and doxorubicin were determined from at least 3 experiments. *P<0.02.

To determine whether disruption in the conversion of hybrid type to complex type N-glycans could affect sensitivity of NB cells to chemotherapeutic drugs, the cell viability of the various NB cell lines treated with and without cisplatin and doxorubicin were detected (Fig 9). In both cases, the drugs were more toxic to the NB_1 cell line than the NB_1(-*Mgat2*) cell line. Taken together, these results support that the faster proliferating NB cell line was more sensitive to cisplatin and doxorubicin than the slower proliferating cell line.

## Discussion

In this study, we provide the first systematic analysis of varied ratios of complex to hybrid types of N-glycans on the transformed phenotype of cultured cancerous cells. It was conducted in NB cell lines that predominantly express either complex type (NB_1), or hybrid type of N-glycans (NB_1(-*Mgat2*)) [19]. We confirmed that complex type N-glycans were attached to proteins when GnT-II was heterologously expressed in the NB_1(-*Mgat2*) cell line, indicating that differences in the N-glycan types of NB cell lines were due to *Mgat2* expression. Interestingly, analysis of the tumorigenic properties of NB cell lines revealed that decreased levels of complex type N-glycans, along with increased levels of hybrid, suppressed colony formation, cell invasiveness, cell growth and proliferation, while it strengthened cell-cell adhesion. Further cell morphology was altered by the type of N-glycans. These experiments suggest that changes in the ratio of complex to hybrid types of N-glycans provide a switch in modifying NB development and progression.

Various N-glycans have been implicated in tumor development and progression. One of the earliest studies linked higher levels of β1,6-branched N-glycans to enhanced metastatic potential [4]. This finding also correlated with progression of neuroblastoma since Hu et al. (2015) [12] observed an increase in several β1,6-branched N-glycan structures in cells (NLF) derived from high-risk neuroblastoma compared to cells (SY5Y) from low-risk neuroblastoma. Additionally, the less aggressive cell line was shown to have increased amounts of hybrid type N-glycans [12]. Our current study shares the role of these two types of N-glycans in neoplastic transformation since lower and higher levels of complex and hybrid types, respectively, decreased cell proliferation and invasiveness, and strengthened cell-cell adhesion. On the contrary, it was reported that increased *Mgat5* (encodes GnT-V, an enzyme that produces β1,6-branched N-glycans) expression levels are associated with favorable neuroblastomas since decreases in *Mgat5* expression levels were associated with more invasive tumor stages [11]. Feasible explanations of these differences could be that the expression level of *Mgat5* is unrepresentative of the amount of β1,6-branched N-glycans, or perhaps tumor cell lines versus cancerous tissue. It remains to be determined whether a higher ratio of β1,6-branched N-glycans to hybrid N-glycans in cancerous tissue, relative to that in normal tissue, promotes NB development and progression.

Since β1,6-branched N-glycans were associated with metastatic potential, swainsonine was used in clinical trials for the treatment of advanced human malignancies [6, 7, 9]. Swainsonine is an indolizidine alkaloid, which inhibits the conversion of hybrid to complex types of N-glycans and thereby lowers the level of β1,6-branched N-glycans [5]. At the cellular level, swainsonine was shown to slow hepatoma cell growth, and decrease cell viability [8]. Our current study was in agreement with a decreased ratio of complex to hybrid types of N-glycans in lowering cell growth by hindering the progression of the cells through the G1+Go phase of the cell cycle, but lacks support for altering cell viability. Another action of swainsonine is to inhibit the lysosomal acid α-mannosidase II [26]. Therefore, it seems reasonable to suspect that the lowered cell viability may be due to inhibition of lysosomal acid α-mannosidase II.

An assigned role of N-glycans, which are often covalently linked to transmembrane proteins [27], is to impact plasma membrane architecture [16–19, 21, 28] and thereby cell signaling events. Growth factor receptors are initiators in the cell signaling cascade for cell differentiation and proliferation [29]. Recently, Anaplastic Lymphoma Kinase (ALK), a receptor kinase involved in pro-survival signaling, was shown to be upregulated in advanced/metastatic neuroblastomas [13, 14]. Further, inhibition of N-glycosylation in ALK-positive neuroblastoma cells, via tunicamycin treatment, disrupted the pro-survival signaling pathway [15]. Herein, we demonstrated that altering the ratio of complex to hybrid types of N-glycans slowed cell growth and proliferation, and changed cell morphology. It is plausible that changes in the type of N-glycans would modify the activity of ALK and thereby the pro-survival signaling pathway.

## Conclusions

Modifications in N-glycans at the cell surface are a dynamic process in the development and progression of a tumor [30, 31], and therefore it is likely that a given set of N-glycan types expressed at the cell surface will modify cancer development and progression. Specifically, we propose that a decline in the ratio of complex to hybrid types of N-glycans will suppress cell proliferation, and cell dissociation and invasive stages of NB.

## Abbreviations

ALK: Anaplastic Lymphoma Kinase
BrdU: 5-bromo-29 deoxyuridine
CHO: Chinese hamster ovarian
E-PHA: *Phaseolus vulgaris* Erythoagglutinin
GlcNAcβ1-*N*-Asn: *N*-acetylglucosamine-β1-*N*-asparagine
GnT: *N*-acetylglucosaminyltransferase
L-PHA: *Phaseolus vulgaris* Leucoagglutinin
NB: Neuroblastoma
